# Pronounced Mitral Annular Disjunction Is Associated With Increased Postoperative Palpitations After Mitral Valve Surgery for Barlow’s Disease

**DOI:** 10.1093/icvts/ivag104

**Published:** 2026-04-10

**Authors:** Martin Bichler, Keti Vitanova, Nina Feirer, Gertrud Goppel, Karin Steiner, Markus Krane, Johannes Boehm

**Affiliations:** Department of Cardiovascular Surgery, Institute INSURE, TUM University Hospital German Heart Center, TUM School of Medicine and Health, Technical University of Munich (TUM), German Heart Center, Lazarettstraße 36, 80636 Munich, Bavaria, Germany; Department of Cardiovascular Surgery, Institute INSURE, TUM University Hospital German Heart Center, TUM School of Medicine and Health, Technical University of Munich (TUM), German Heart Center, Lazarettstraße 36, 80636 Munich, Bavaria, Germany; Department of Cardiovascular Surgery, Institute INSURE, TUM University Hospital German Heart Center, TUM School of Medicine and Health, Technical University of Munich (TUM), German Heart Center, Lazarettstraße 36, 80636 Munich, Bavaria, Germany; Department of Cardiovascular Surgery, Institute INSURE, TUM University Hospital German Heart Center, TUM School of Medicine and Health, Technical University of Munich (TUM), German Heart Center, Lazarettstraße 36, 80636 Munich, Bavaria, Germany; Department of Cardiovascular Surgery, Institute INSURE, TUM University Hospital German Heart Center, TUM School of Medicine and Health, Technical University of Munich (TUM), German Heart Center, Lazarettstraße 36, 80636 Munich, Bavaria, Germany; Department of Cardiovascular Surgery, Institute INSURE, TUM University Hospital German Heart Center, TUM School of Medicine and Health, Technical University of Munich (TUM), German Heart Center, Lazarettstraße 36, 80636 Munich, Bavaria, Germany; DZHK (German Center for Cardiovascular Research), Partner Site Munich Heart Alliance, Lazarettstraße 36, 80636 Munich, Bavaria, Germany; Department of Cardiovascular Surgery, Institute INSURE, TUM University Hospital German Heart Center, TUM School of Medicine and Health, Technical University of Munich (TUM), German Heart Center, Lazarettstraße 36, 80636 Munich, Bavaria, Germany

**Keywords:** mitral valve, mitral valve surgery, mitral annular disjunction, Barlow’s disease, palpitations

## Abstract

**Objectives:**

Pronounced mitral annular disjunction (pMAD ≥8 mm) in myxomatous mitral valve disease has been associated with ventricular arrhythmias in selected cohorts, whereas minor degrees of mural separation are frequently observed in structurally normal hearts. Its clinical relevance after mitral valve surgery remains unclear. We investigated whether pMAD is associated with postoperative symptom burden and rhythm outcomes.

**Methodsv:**

In this retrospective cohort with prospective follow-up, 246 adults undergoing mitral valve surgery for Barlow’s disease were classified intraoperatively as pMAD (≥8 mm) or non-pMAD (<8 mm). The primary end point was patient-reported postoperative palpitations. Propensity score–based inverse probability weighting was applied to adjust for baseline differences. Structured follow-up in pMAD patients included transthoracic echocardiography (TTE) and 24-hour Holter monitoring.

**Results:**

pMAD was present in 103 patients (41.9%). Surgery effectively reduced disjunction distance. At follow-up, palpitations were more frequent in pMAD compared with non-pMAD patients (40.8% vs 18.9%, *P* = .011), remaining significant after adjustment. In the pMAD follow-up cohort, atrial fibrillation prevalence decreased significantly after surgery. Holter monitoring did not demonstrate sustained malignant arrhythmias. Survival and major adverse events were comparable between groups.

**Conclusions:**

After mitral valve surgery for Barlow’s disease, pMAD is independently associated with increased postoperative palpitations despite anatomical correction. This association was not accompanied by excess malignant arrhythmias or adverse clinical end points, suggesting persistent symptom susceptibility rather than overt electrical instability.

## INTRODUCTION

Mitral annular disjunction (MAD) is a structural abnormality characterized by a separation between the left atrial-mitral valve annulus and the left ventricular myocardium.^1^ It is predominantly observed in patients with Barlow’s disease and mitral valve prolapse^2^^,^^3^ and has garnered increasing attention due to its potential link to arrhythmic events and sudden cardiac death.^4–6^ In the present study, we specifically investigated pronounced mitral annular disjunction (pMAD), defined as a separation of ≥8 mm between the atrial wall–mitral leaflet hinge point and the left ventricular myocardial crest. For clarity, throughout this manuscript, the term pMAD refers exclusively to this phenotype. Prior studies suggest that pronounced forms of MAD in selected patients with mitral valve prolapse may be associated with ventricular arrhythmias and sudden cardiac death.^3–5^ Recent data indicate that minor degrees of mural separation between the atrial and ventricular myocardium may also be encountered in structurally normal hearts, suggesting that MAD represents a continuous rather than dichotomous anatomical spectrum.^7^ Prior studies have used heterogeneous definitions of “mitral annular disjunction,” and minor mural atrioventricular separation appears common in healthy individuals. We therefore focused exclusively on a pre-specified pronounced phenotype (≥8 mm) in Barlow’s disease to distinguish potentially pathological variants from physiological findings. Toh et al. demonstrated that up to 90% of healthy individuals exhibit some degree of mural separation, most often in commissural regions of the annulus. Beyond its structural displacement, pronounced disjunction in myxomatous mitral valve disease may impair mechano–electrical coupling at the atrioventricular junction, promoting myocardial stretch and fibrosis, which can persist and contribute to an arrhythmic substrate even after anatomical correction.^8^^,^^9^ Although an association between pronounced disjunction in degenerative mitral valve disease and ventricular arrhythmias has been reported,^9^^,^^10^ its effect on postoperative symptom burden and patient-reported outcomes after mitral valve surgery remains largely unexplored.^1^^,^^3–5^ Palpitations often occur in patients with mitral valve disease, both before and after surgical intervention,^11^ and may significantly impair patients’ quality of life.^12^ However, it is unclear whether pMAD contributes independently to postoperative symptoms despite successful surgical correction of the mitral valve and apparent anatomical normalization of the annular geometry.^13^ The present study investigated the clinical relevance of pMAD for postoperative symptom burden.

## METHODS

### Study design and population

**Figure 1.**
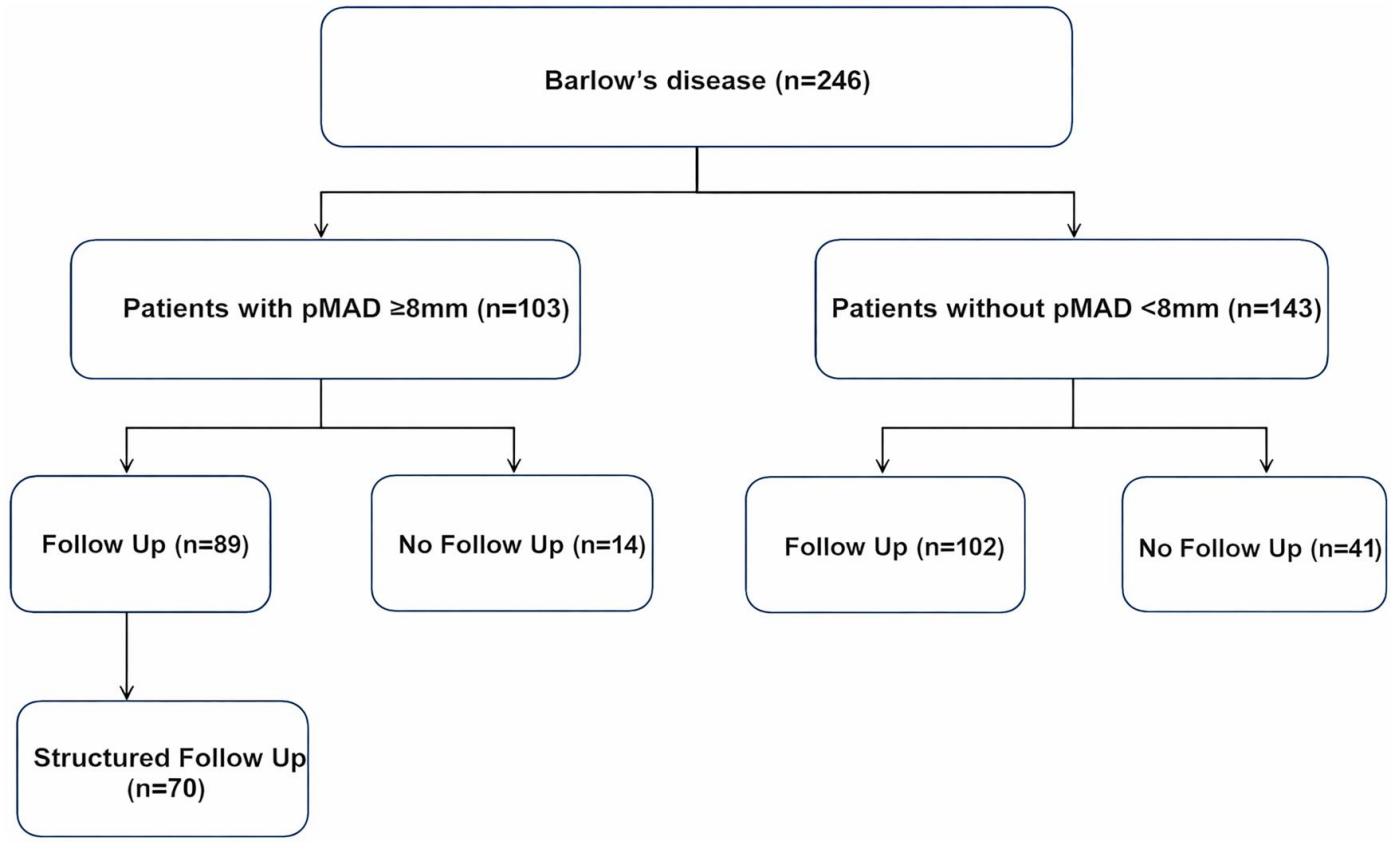
Patient Flow Chart.

Adult patients with Barlow’s disease undergoing mitral valve surgery at the German Heart Center between 2014 and 2023 were included in this single-centre retrospective cohort with prospective follow-up (Figure 1). Prior mitral valve surgery and infective endocarditis were exclusion criteria.

**Figure:**
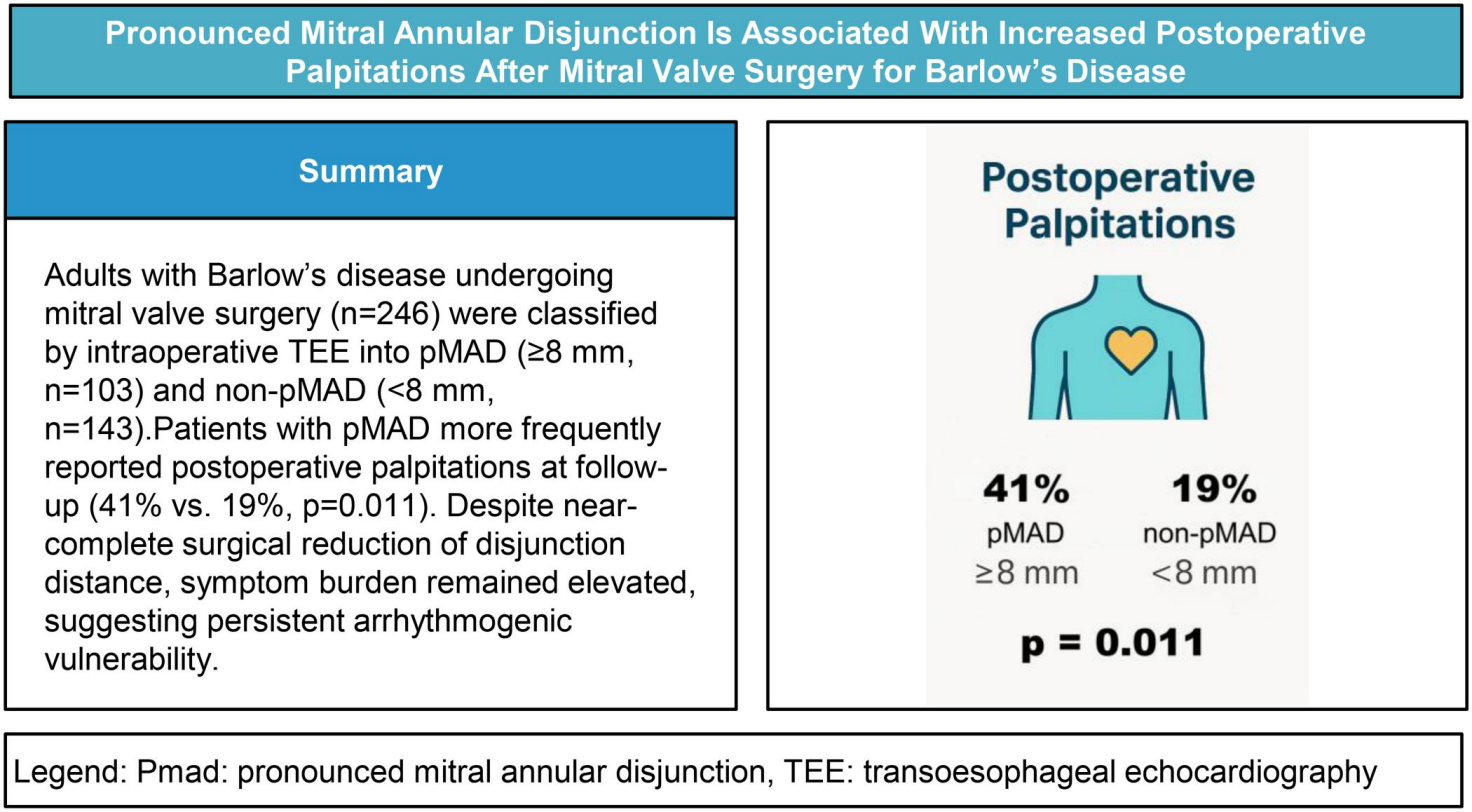


### Pronounced MAD assessment

We defined pMAD as a separation of ≥8 mm between the atrial wall–mitral leaflet hinge junction and the crest of the left ventricular myocardium, measured in the mid-oesophageal intercommissural view at end-systole using intraoperative transoesophageal echocardiography. Representative measurements are shown in Figure S1. Patients were stratified into pMAD (≥8 mm) and non-pMAD (<8 mm), based on prior data linking marked disjunction to increased arrhythmic risk.^1^^,^^3–5^^,^^14–16^

### Follow-up assessment for all patients

All patients received a standardized follow-up questionnaire (EQ-5D including VAS 0-100). Non-responders were contacted by structured telephone interviews capturing the same core items.

### Follow-up assessment in pMAD subgroup

Structured follow-up including transthoracic echocardiography (TTE) and 24-hour Holter ECG was performed exclusively within the pMAD subgroup. Holter recordings were obtained at variable postoperative time points rather than at a predefined standardized interval and should therefore be interpreted as cross-sectional rhythm assessments. Non-pMAD patients did not undergo systematic Holter monitoring as part of the study protocol. Echocardiography assessed pMAD distance, left atrial and left ventricular end-diastolic diameters, left ventricular ejection fraction, and residual insufficiency. Holter recordings were analysed for atrial and ventricular arrhythmias. Analyses were performed including and excluding patients undergoing concomitant surgical atrial fibrillation ablation (MAZE).

### Study end points

The primary end point was binary patient-reported palpitations at follow-up, defined as subjective irregular or forceful heartbeats occurring after surgery; analyses were limited to prevalence due to the absence of onset timing. Secondary end points comprised all-cause mortality, major adverse cardiovascular events, mitral valve reoperation, atrial fibrillation at follow-up, and EQ-5D-assessed health status. Clinical definitions followed ESC guidelines, with diabetes mellitus as a documented diagnosis or use of antidiabetic medication.

### Statistical analysis

Statistical analyses were performed using IBM SPSS Statistics (version 31.0.0.0). Continuous variables are presented as mean ± SD and compared using Student’s *t*-test or ANOVA after Shapiro–Wilk testing. Categorical variables are reported as counts and percentages and compared using the *χ*^2^ test. Kaplan–Meier analyses with log-rank testing were applied to time-dependent secondary end points (all-cause mortality, major adverse cardiovascular events, and reoperation). Palpitations were analysed as a binary patient-reported outcome at follow-up; time-to-event analyses were not performed due to the absence of reliable symptom onset timing. To account for baseline imbalances, a propensity score for pMAD was estimated using multivariable logistic regression including age, sex, arterial hypertension, preoperative atrial fibrillation, and concomitant MAZE procedure. Inverse probability weighting with stabilized weights was applied, and covariate balance was assessed using standardized mean differences. Missing baseline data were rare (<5%) and handled by case-wise exclusion. Echocardiographic parameters, available only in the pMAD subgroup, were excluded from the primary model. Sensitivity analyses were performed within the pMAD follow-up cohort, including echocardiographic variables. The primary exposure was pMAD (≥8 mm); distance per millimetre was analysed exploratorily. Non-linear age effects were assessed using a quadratic term. A 2-sided *P* < .05 was considered statistically significant.

## RESULTS

### Patient characteristics

Baseline demographic and perioperative characteristics of the study cohort are summarized in Table 1. A total of 246 patients with Barlow’s disease were included in the study. Of these, 191 patients (77.6%) provided follow-up data, either by returning the standardized questionnaire (*n* = 166) or via a structured telephone interview (*n* = 25). In our cohort, pMAD was present in 103 patients (41.9%). Patients lost to follow-up had a significantly lower prevalence of pMAD (23.6% vs 47.1%, *P* = .002; Table S1). In the overall cohort, the mean age at surgery was 56.6 ± 12.5 years, and 67.1% of patients were male. The majority of patients underwent mitral valve repair (90.7%). For analyses comparing outcomes between patients with and without pMAD, inverse probability of treatment weighting was applied, resulting in well-balanced baseline characteristics between groups (all standardized mean differences <0.1). No evidence for a non-linear association between age and postoperative palpitations was observed (quadratic age term *P* = .203).

**Table 1.** Baseline Patient Characteristics

	All patients (*n* = 246)	pMAD (*n* = 103)	Non-pMAD (*n* = 143)	*P*-value
**Demographic characteristics**				
Male gender	165 (67.1%)	68 (66.0%)	97 (67.8%)	.435^a^
Age at surgery (years)	56.6 ± 12.5	55.0 ± 12.4	56.1 ± 12.6	.510^b^
Height (cm)	176.41 ± 9.6	177.26 ± 9.9	175.8 ± 9.3	.240^b^
Weight (kg)	75.0 ± 14.7	74.7 ± 14.0	75.2 ± 15.3	.821^b^
**Operative data**				
MVr	223 (90.7%)	95 (92.2%)	128 (89.5%)	.311^a^
MVR	23 (9.3%)	8 (7.8%)	15 (10.5%)	.311^a^
±TVr	44 (17.9%)	17 (16.5%)	27 (18.9%)	.380^a^
±MAZE	27 (11.0%)	9 (8.7%)	18 (12.6%)	.229^a^
±AVr/AVR	9 (3.7%)	3 (2.9%)	6 (4.2%)	.434^a^
±CABG	13 (5.3%)	4 (3.9%)	9 (6.3%)	.297^a^
**Pre-existing conditions**				
MAD distance (mm)	6.3 ± 3.8	10.1 ± 1.8	3.7 ± 2.4	**<.001** ^b^
Arterial hypertension	115 (46.7%)	41 (39.8%)	74 (51.7%)	**.042** ^a^
Hyperlipidemia	80 (32.5%)	36 (35.0%)	44 (30.8%)	.290^a^
Diabetes	4 (1.6%)	1 (1.0%)	3 (2.1%)	.442^a^
Positive family history	67 (27.2%)	28 (27.2%)	39 (27.3%)	.528^a^
Smoking	76 (30.9%)	32 (31.1%)	44 (30.8%)	.535^a^
Patient-reported palpitations preoperative	47 (19.1%)	20 (19.4%)	27 (18.9%)	.547^a^
**Follow-up**				
Existing FU	191 (77.6%)	89 (86.4%)	102 (71.3%)	
Time to follow-up (years)	3.2 ± 2.3	3.1 ± 2.2	3.2 ± 2.4	.686^b^
MACE (myocardial infarction, stroke)	11 (4.4%)	6 (5.8%)	5 (3.5%)	.293^a^
Mortality	1 (0.5%)	1 (1.0%)	0 (0%)	.466^a^
Reoperation	8 (4.2%)	4 (3.9%)	4 (2.8%)	.562^a^
EQ-5D VAS (0-100)	78.4 ± 1.5	76.7 ± 19.9	79.8 ± 15.8	.311^b^
Patient-reported palpitations at Follow-up	69 (28.0%)	42 (40.8%)	27 (18.9%)	**.011** ^a^
Beta-blocker intake	75 (30.5%)	37 (35.9%)	38 (26.6%)	.156^a^
Oral anticoagulation therapy	63 (25.6%)	25 (24.3%)	38 (26.6%)	.173^a^
Implantation of an implantable cardioverter-defibrillator or pacemaker	8 (3.2%)	1 (1.0%)	7 (4.9%)	.058^a^

Values are mean ± SD or *n* (%).

a *χ*² test.

b *t*-test or ANOVA.

Abbreviations: AVr/AVR = aortic valve repair/replacement, CABG = coronary artery bypass grafting, FU = follow-up, ICD = implantable cardioverter-defibrillator, MACE = major adverse cardiac events (myocardial infarction, stroke), MAZE = surgical ablation procedure for atrial fibrillation, MVr = mitral valve repair, MVR = mitral valve replacement, SD = standard deviation, TVr = tricuspid valve repair. Bold values are  statistically significant.

### Primary end point: palpitations

**Figure 2.**
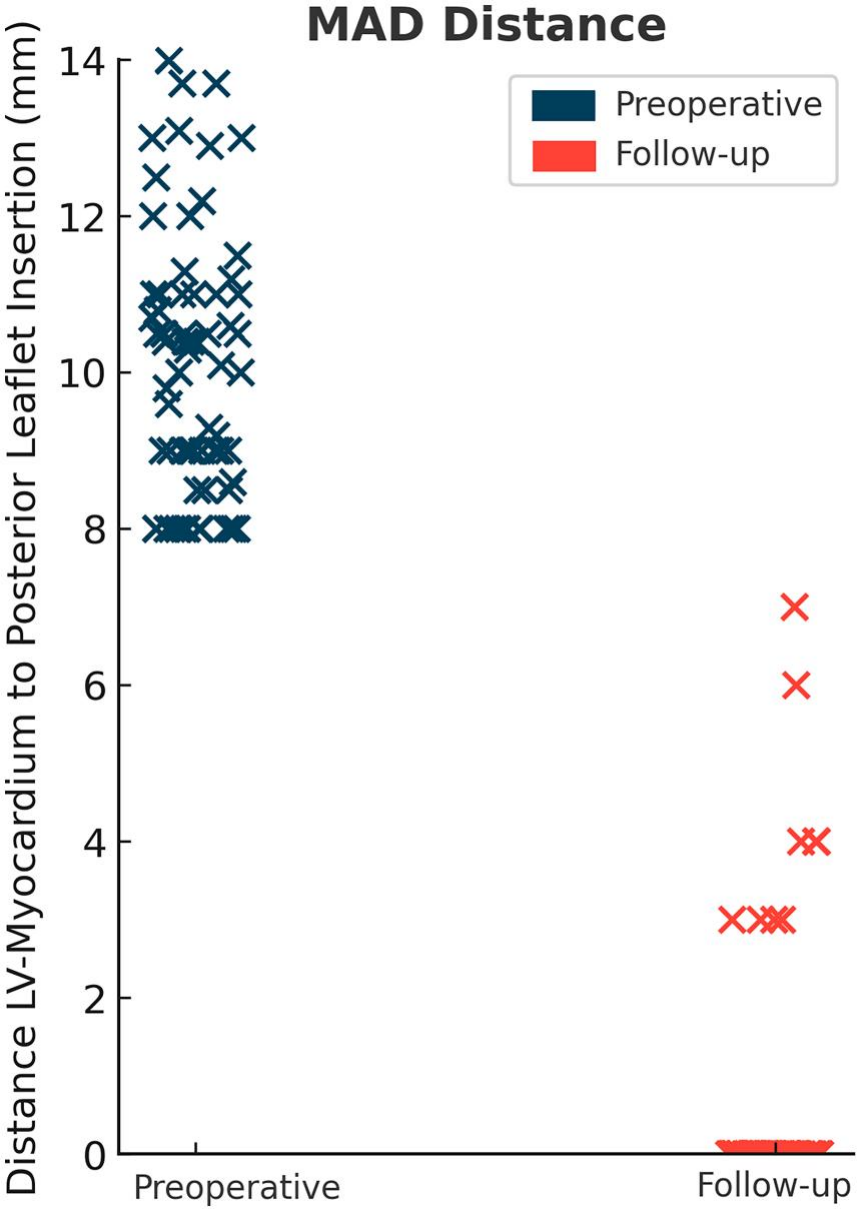
MAD Distance Before and After Mitral Valve Surgery in pMAD Patients (*n* = 70).

Patient-reported palpitations were more frequent in patients with pMAD than in those without (40.8% vs 18.9%, *P *= .011). After inverse probability weighting, pMAD remained independently associated with postoperative palpitations (OR 3.07, 95% CI, 1.72-5.47, *P *< .001). This association persisted despite successful surgical correction, as reflected by a marked reduction in pMAD distance from 10.2 ± 1.8 to 0.5 ± 1.5 mm (Figure 2). In multivariable logistic regression, age (OR 1.03, 95% CI, 1.00-1.07, *P *= .027), MAZE procedure (OR 3.25, 95% CI, 1.16-9.07, *P *= .024), and MAD distance (OR 1.09, 95% CI, 1.00-1.18, *P *= .047) were independently associated with postoperative palpitations (Table 2).

**Table 2.** Sensitivity Analysis of Postoperative Palpitations Using Continuous MAD Distance

Variable	Univariable OR (95% CI)	*P*-value	Multivariable OR (95% CI)	*P*-value
Age (per year)	1.037 (1.009-1.066)	.009	1.03 (1.004-1.065)	.027
Male sex	0.683 (0.382-1.219)	.197	0.633 (0.322-1.246)	.186
Preoperative AF	0.582 (0.287-1.180)	.133	0.510 (0.235-1.106)	.088
MAZE procedure	2.274 (1.005-5.146)	.049	3.249 (1.164-9.065)	.024
MAD distance (per mm)	1.083 (1.006-1.167)	.035	1.088 (1.001-1.182)	.047

Model performance: *χ*² = 20.654 (*P* < .001); Nagelkerke *R*² = 0.140; Hosmer–Lemeshow *P* = .879; overall classification = 67%.

Abbreviations: AF = atrial fibrillation, MAD = mitral annular disjunction, MAZE = surgical ablation procedure for atrial fibrillation.

The MAZE coefficient likely reflects confounding by indication, as rhythm-symptomatic patients were preferentially selected for ablation.

### Secondary end points

**Figure 3.**
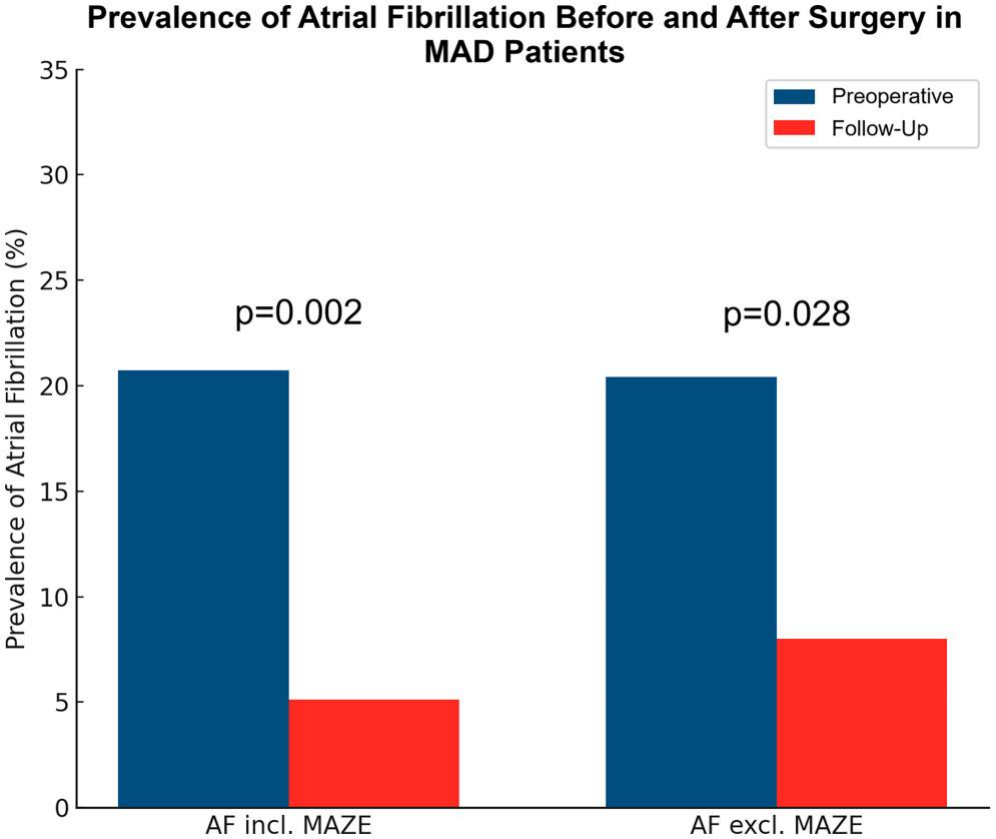
Prevalence of Atrial Fibrillation Before and After Surgery in pMAD Patients.

No significant differences were observed between groups in all-cause mortality, major adverse cardiovascular events, or mitral valve reoperation. Self-perceived health status was comparable between patients with and without pMAD. In the structured pMAD follow-up cohort, Holter ECG demonstrated a significant reduction in atrial fibrillation prevalence from preoperative to postoperative assessment, from 27.9% preoperatively to 7.4% at follow-up (Figure 3).

### Echocardiographic follow-up in pMAD patients

In total, 70 pMAD patients completed structured echocardiographic follow-up. MAD distance markedly decreased from 10.2 ± 1.8 mm preoperatively to 0.5 ± 1.5 mm postoperatively (*P *< .001; Table 3, Figure 2). Significant reductions were also observed in LVEDD (5.2 ± 0.8 to 4.4 ± 0.5 cm), left atrial diameter (4.5 ± 0.7 to 3.7 ± 0.7 cm), and mitral regurgitation grade (3.0 ± 0.0 to 1.1 ± 0.6; all *P *< .001). LVEF showed a small numerical decrease (59.3 ± 5.3% to 57.2 ± 6.9%, *P *= .025) while remaining within the normal range. In multivariable analyses within the pMAD follow-up cohort, none of the echocardiographic parameters were independently associated with postoperative palpitations, and their inclusion did not materially alter the primary results (Table S2).

**Table 3.** pMAD Follow-Up *n* = 70

	Preoperative	Follow-up	*P*-value
**Echocardiogram: *n* = 70**			
MAD distance	10.2 ± 1.8	0.5 ± 1.5	**<.001** ^a^
LVEDD	5.2 ± 0.8	4.4 ± 0.5	**<.001** ^a^
LA diameter	4.5 ± 0.7	3.7 ± 0.7	**<.001** ^a^
LVEF	59.3 ± 5.3	57.2 ± 6.9	**.025** ^a^
Mitral valve insufficiency (degree 0-3)	3.0 ± 0	1.1 ± 0.6	**<.001** ^a^
Tricuspid valve insufficiency (degree 0-3)	1.0 ± 0.7	0.8 ± 0.5	.078^a^
NYHA (degree 1-4)	2.14 ± 0.7	1.36 ± 0.6	**<.001** ^a^
**Holter-ECG: *n* = 68**			
Atrial fibrillation (incl. MAZE) *n* = 68	19 (27.9%)	5 (7.4%)	**.002** ^a^
Atrial fibrillation (excl. MAZE) *n* = 63	14 (22.2%)	5 (7.9%)	**.028** ^a^

Values are mean ± SD.

a *T* test for samples with paired values.

Abbreviations: ECG = electrocardiogram, LA = left atrium, LVEDD = left ventricular end-diastolic diameter, LVEF = left ventricular ejection fraction, MAD = mitral annular disjunction, MAZE = surgical ablation procedure for atrial fibrillation, NYHA = New York Heart Association, SD = standard deviation. Bold values are statistically significant.

### Holter ECG follow-up in pMAD patients

In the pMAD follow-up cohort, Holter ECG data were available in 68 of 70 patients. Preoperatively, 19 of 68 (27.9%) had atrial fibrillation, decreasing to 5 of 68 (7.4%) at follow-up (*P* = .002). After excluding patients undergoing concomitant surgical AF ablation (MAZE), AF prevalence declined from 14 of 63 (22.2%) to 5 of 63 (7.9%) (*P* = .028) (Figure 3). Within the pMAD follow-up cohort, no sustained malignant arrhythmias were documented that would clearly account for the overall prevalence of patient-reported palpitations. Because Holter monitoring and symptom assessment were not performed simultaneously, no formal symptom–rhythm correlation analysis was possible, underscoring the dissociation between subjective symptoms and documented rhythm disturbances.

## DISCUSSION

In this retrospective cohort with prospective follow-up, pMAD was linked to a higher prevalence of postoperative palpitations. This association remained robust after adjustment for baseline differences using propensity score–based inverse probability weighting, supporting an independent relationship between pronounced disjunction and postoperative symptom burden. Importantly, palpitations represent a patient-reported symptom rather than a direct measure of arrhythmic burden and should therefore not be equated with sustained or malignant arrhythmias. This finding persisted despite successful anatomical correction of the disjunction during surgery, as reflected by a marked reduction in MAD distance from 10.2 ± 1.8 mm preoperatively to 0.5 ± 1.5 mm on follow-up TTE. Recent population-imaging data demonstrate that mural atrioventricular separation may also be observed in structurally normal hearts, with clinically relevant inferolateral disjunction being rare. Accordingly, our study focused on pronounced disjunction associated with myxomatous mitral valve disease to distinguish pathological variants from physiological findings.^17^ These findings are consistent with persistent symptom susceptibility and residual substrate, although our data do not establish a causal rhythm mechanism.^3^^,^^4^^,^^18^ A recent meta-analysis reported an association between MAD and complex ventricular arrhythmias, even in patients without left ventricular dysfunction.^19^ However, given that minor mural separation is frequently observed in structurally normal hearts, these findings likely reflect selected phenotypes with more pronounced disjunction and concomitant myxomatous disease. Notably, the reduction in atrial fibrillation prevalence was observed even in patients who did not undergo concomitant ablation, further supporting the hypothesis of partial rhythm stabilization following mitral valve surgery alone.^8^

Our findings align with prior studies suggesting a potential arrhythmogenic relevance of pronounced disjunction in selected patients with mitral valve prolapse, particularly in the context of mitral valve prolapse (MVP).^2–4^^,^^20–22^ While existing literature predominantly focuses on sudden cardiac death or complex ventricular arrhythmias, our study extends this evidence by linking pMAD to persistent postoperative palpitations.^23^ Logistic regression further identified MAD distance as an independent predictor, indicating an incremental increase in palpitations with greater disjunction extent.^19^^,^^24–26^ The observed postoperative reduction in atrial fibrillation prevalence supports effective rhythm stabilization and suggests that persistent palpitations are not solely attributable to recurrent atrial fibrillation but may reflect residual electrical or perceptual mechanisms. Despite a marked reduction of disjunction distance and a decline in atrial fibrillation prevalence, 40.8% of pMAD patients continued to report palpitations without documented sustained malignant arrhythmias. Although structured Holter monitoring in the pMAD subgroup did not demonstrate an excess of sustained malignant arrhythmias, postoperative palpitations remained frequent. Given the absence of simultaneous rhythm recording and symptom documentation, a direct causal relationship between documented arrhythmias and reported palpitations cannot be established. Several non-exclusive mechanisms may explain this dissociation. Palpitations may reflect intermittent ectopy or short-lived supraventricular or ventricular rhythm disturbances not captured during a single 24-hour recording. In addition, benign arrhythmias below conventional “malignant” thresholds or heightened symptom perception may contribute. Because Holter monitoring and symptom assessment were not temporally synchronized, a direct symptom–rhythm correlation cannot be established. A relevant proportion of patients had documented preoperative atrial fibrillation, and not all underwent concomitant surgical ablation, which may have contributed to postoperative symptom reporting. The persistence of palpitations in a substantial proportion of pMAD patients despite anatomical correction supports the concept of pMAD as a structural–electrical interface, potentially contributing to residual electrical instability or heightened symptom perception.^5^^,^^13^^,^^27^ Consistent with this hypothesis, cardiac MRI studies have demonstrated LGE-positive fibrosis in the inferolateral wall and papillary muscles, regions frequently affected by MAD, which is independently associated with ventricular arrhythmias and sudden cardiac death in patients with mitral valve prolapse.^26^^,^^28^ These observations may help explain persistent palpitations even in the absence of sustained arrhythmias.^28^ To address potential confounding by concomitant rhythm interventions, atrial fibrillation prevalence was analysed both including and excluding patients undergoing MAZE procedures. The association between MAZE and postoperative palpitations likely reflects confounding by indication, as rhythm-symptomatic patients were preferentially selected for ablation.^29^^,^^30^ Importantly, even after exclusion of MAZE patients, atrial fibrillation prevalence was significantly reduced, suggesting partial rhythm stabilization attributable to mitral valve surgery itself, potentially via mechanical or structural remodelling of the annular–atrial interface.^31^^,^^32^ From a pathophysiological perspective, pMAD in degenerative mitral valve disease has been associated with altered mechanical coupling between the atrial and ventricular myocardium, potentially promoting arrhythmogenic mechanisms.^1–4^^,^^20^^,^^33^ Our findings suggest that this association may persist despite anatomical correction, manifesting primarily as postoperative symptom burden. Importantly, no differences in mortality, MACE, or reoperation rates were observed. This indicates that pronounced MAD does not affect survival or structural durability but rather postoperative symptoms.^14^^,^^25^^,^^34^ Palpitations were assessed as a binary patient-reported outcome without recording an exact onset date; therefore, analyses were limited to prevalence-based comparisons and logistic regression rather than time-to-event modelling. Although palpitations were significantly more prevalent among pMAD patients, self-perceived health status did not differ between groups.^35^ This underscores the distinction between rhythm-related symptom burden and overall subjective well-being. Persistent palpitations may not necessarily impair overall health perception.^36^ Despite their subjective nature, the consistent reporting of palpitations supports their relevance as a patient-reported outcome.

## LIMITATIONS

This study has several limitations. It was a retrospective, single-centre experience, and non-randomized, which may have introduced selection bias. Follow-up completeness differed between pMAD and non-pMAD groups (87.4% vs 70.6%). Given the differential follow-up rates, prevalence estimates may be influenced by attrition bias and should be interpreted with caution. Patients lost to follow-up had a significantly lower prevalence of pMAD, reflecting targeted recruitment of patients with pronounced disjunction for structured follow-up. Missing data were rare (<5%) and handled by case-wise exclusion. Echocardiographic parameters were available only in the pMAD subgroup and were excluded from the overall regression model. Palpitations were assessed by patient report and are therefore subject to recall bias. In addition, palpitations were captured as a binary outcome without detailed characterization regarding frequency, duration, or underlying rhythm. Structured postoperative Holter ECG monitoring was performed exclusively in patients with pMAD and was not systematically obtained in non-pMAD patients. Consequently, direct comparison of arrhythmia burden between groups was not possible within this study. Furthermore, Holter recordings and symptom assessment were not temporally synchronized. Finally, the moderate sample size and limited number of events may have reduced statistical power and prevented detection of smaller effects. Furthermore, the extent of MAD adjacent to the commissures was not systematically assessed. Measurements were primarily performed in the intercommissural view focusing on the posterior annulus, which may underestimate the circumferential extent of disjunction.

## CONCLUSION

In this cohort, pMAD ≥8 mm was independently associated with increased patient-reported postoperative palpitations after mitral valve surgery for Barlow’s disease. Within the structured pMAD follow-up subgroup, systematic 24-hour Holter monitoring did not reveal sustained malignant arrhythmias of a frequency that would account for the overall prevalence of patient-reported palpitations. These findings suggest a symptom-predominant phenotype rather than overt electrical instability. Prospective studies with systematic rhythm monitoring in both groups are required to clarify underlying mechanisms.

## Supplementary Material

ivag104_Supplementary_Data

## Data Availability

The data underlying this article are not publicly available due to privacy and ethical restrictions but can be shared upon reasonable request to the corresponding author and with permission of the institutional review board.

## Abbreviations

AF: atrial fibrillation
AVr/AVR: aortic valve repair/replacement
CABG: coronary artery bypass grafting
ECG: electrocardiogram
EQ-5D: EuroQol 5 dimensions questionnaire
LA: left atrium
LVEDD: left ventricular end-diastolic diameter
LVEF: left ventricular ejection fraction
MACE: major adverse cardiovascular events
MAD: mitral annular disjunction
MAZE: surgical ablation procedure for atrial fibrillation
MVP: mitral valve prolapse
MVr/MVR: mitral valve repair/replacement
NYHA: New York Heart Association
pMAD: pronounced MAD
ROC: receiver operating characteristic
SVT: supraventricular tachycardia
TEE: transoesophageal echocardiography
TTE: transthoracic echocardiography
TVr: tricuspid valve repair
