# Supplementary material for: Pronounced Mitral Annular Disjunction Is Associated With Increased Postoperative Palpitations After Mitral Valve Surgery for Barlow’s Disease
Source: Interdiscip Cardiovasc Thorac Surg. 2026 Apr 10;41(4):ivag104. doi: 10.1093/icvts/ivag104 (PMC13110859; doi:10.1093/icvts/ivag104)
Supplement: ivag104_Supplementary_Data [file ivag104_supplementary_data.zip › Supplementary Table S2. Multivariable logistic regression.docx]

| **Supplementary Table S2.**  **Multivariable logistic regression for postoperative palpitations in patients with pMAD (n = 70)** | | | |
| --- | --- | --- | --- |
| **Variable** | **Odds ratio (OR)** | **95 % CI** | **p-value** |
| Age at surgery (per year) | 1.05 | 0.99–1.11 | 0.108 |
| Sex (male) | 0.64 | 0.17–2.41 | 0.507 |
| MAZE procedure | 0.32 | 0.04–2.80 | 0.304 |
| Preoperative arrhythmia | 0.88 | 0.27–2.86 | 0.825 |
| LVEDD (per cm) | 0.85 | 0.32–2.29 | 0.749 |
| Left atrial diameter (per cm) | 1.37 | 0.63–2.95 | 0.429 |
| LVEF (per %) | 1.04 | 0.96–1.12 | 0.341 |

Multivariable logistic regression including clinical and echocardiographic variables. Odds ratios are shown per unit increase for continuous variables. This analysis was performed as an exploratory sensitivity analysis performed due to availability of echocardiographic parameters only in the pMAD subgroup.
