# Supplementary material for: Pronounced Mitral Annular Disjunction Is Associated With Increased Postoperative Palpitations After Mitral Valve Surgery for Barlow’s Disease
Source: Interdiscip Cardiovasc Thorac Surg. 2026 Apr 10;41(4):ivag104. doi: 10.1093/icvts/ivag104 (PMC13110859; doi:10.1093/icvts/ivag104)
Supplement: ivag104_Supplementary_Data [file ivag104_supplementary_data.zip › Supplementary Figure S1.docx]

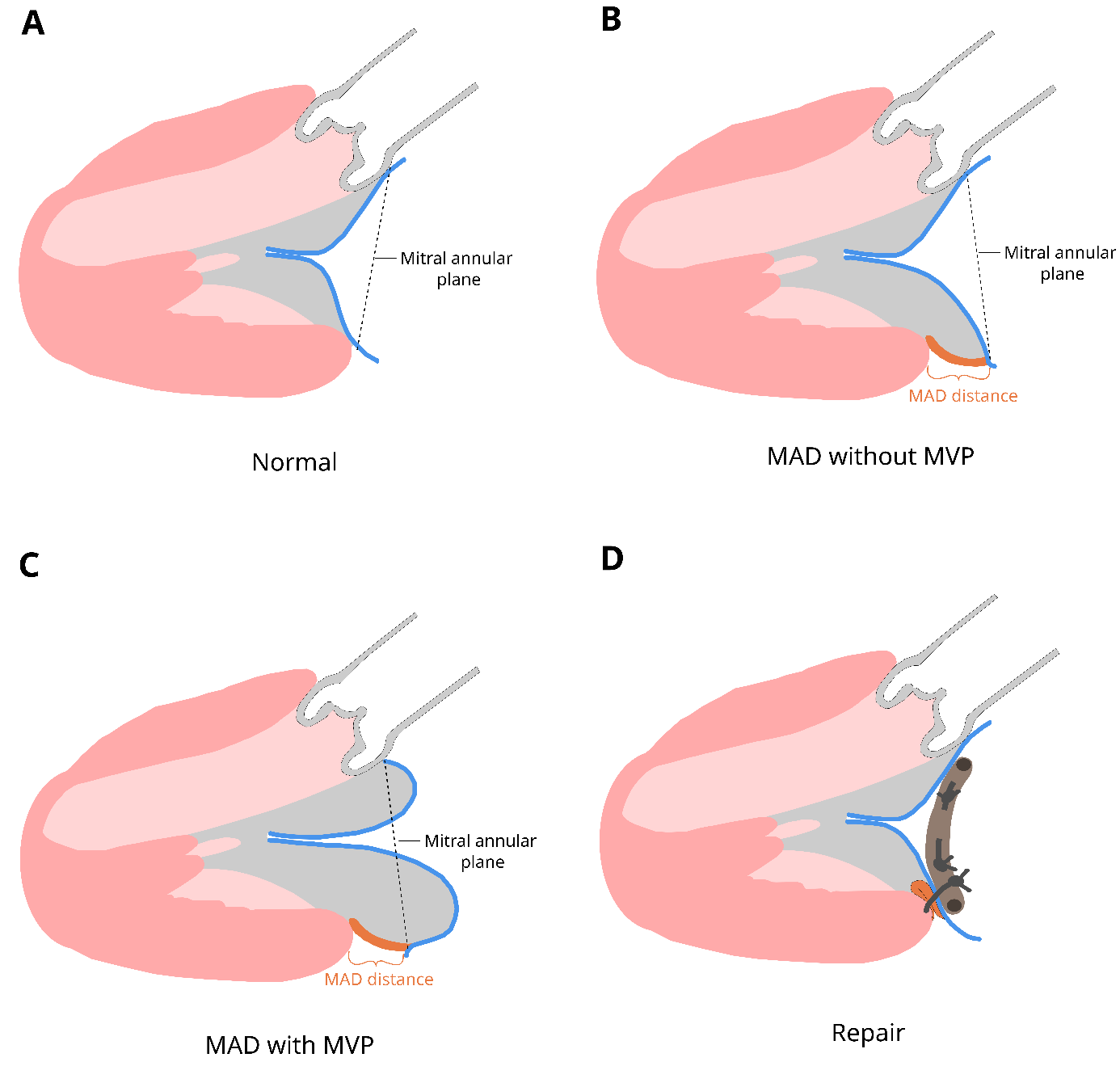


Supplementary Figure S1. Illustration of pronounced mitral annular disjunction and its surgical correction.

Panel A depicts normal mitral valve anatomy with close alignment of the atrial wall–leaflet hinge junction and the left ventricular myocardium.

Panel B illustrates mitral annular disjunction without mitral valve prolapse, highlighting the separation between the atrial wall–leaflet hinge junction and the ventricular myocardium.

Panel C shows pronounced disjunction in the presence of mitral valve prolapse.

Panel D demonstrates reduction of the disjunction following mitral valve repair.

For consistency, the dashed line indicates the geometric reference plane used for measurement rather than implying a discrete anatomical annulus in the presence of pronounced disjunction.
