# Supplementary material for: Pronounced Mitral Annular Disjunction Is Associated With Increased Postoperative Palpitations After Mitral Valve Surgery for Barlow’s Disease
Source: Interdiscip Cardiovasc Thorac Surg. 2026 Apr 10;41(4):ivag104. doi: 10.1093/icvts/ivag104 (PMC13110859; doi:10.1093/icvts/ivag104)
Supplement: ivag104_Supplementary_Data [file ivag104_supplementary_data.zip › Supplementary Table S1. BC with and without FU.docx]

| **Supplement Table S1. Comparison of baseline characteristics between patients with and without follow-up** | | | | |
| --- | --- | --- | --- | --- |
| **Variable** | **Follow-up available (n=191)** | | **Lost to follow-up (n=55)** | **p-value** |
| Age at surgery (mean ±SD) | | 55.9 ±11.9 | 54.2 ±14.9 | 0.374 |
| Male sex (%) | | 67.5 (129/191) | 63.6 (35/55) | 0.588 |
| pMAD ≥ 8 mm(%) | | 47.1 (90/191) | 23.6 (13/55) | **0.002** |
| Hypertension (%) | | 49.7 (95/191) | 36.3 (20/55) | 0.080 |
| Preoperative AF (%) | | 25.1 (48/191) | 20.0 (11/55) | 0.432 |
| MV repair (%) | | 92.7 (177/191) | 83.6 (46/55) | **0.043** |
| MAZE procedure (%) | | 10.0 (19/191) | 12.7 (7/55) | 0.563 |
| **Abbreviations:** pMAD = pronounced mitral annular disjunction; AF = atrial fibrillation; MV = Mitral valve. All comparisons performed by χ² test for categorical variables and independent-samples t-test for continuous variables. P-values are unadjusted and intended to describe follow-up availability rather than causal associations. Patients lost to follow-up had a significantly lower prevalence of pMAD. Minor differences were also observed in mitral valve repair versus replacement rates. Apart from pMAD prevalence and mitral valve repair versus replacement rates, other baseline characteristics were broadly comparable between patients with and without available follow-up. | | | | |
